# Identification of protoplast-isolation responsive microRNAs in *Citrus reticulata* Blanco by high-throughput sequencing

**DOI:** 10.1371/journal.pone.0183524

**Published:** 2017-08-22

**Authors:** Xiaoyong Xu, Xiaoling Xu, Yipeng Zhou, Shaohua Zeng, Weiwen Kong

**Affiliations:** 1 School of Horticulture and Plant Protection, Yangzhou University, Yangzhou, China; 2 Joint International Research Laboratory of Agriculture and Agri-Product Safety of the Ministry of Education, Yangzhou University, Yangzhou, China; 3 Guangdong Provincial Key Laboratory of Applied Botany, South China Botanical Garden, Chinese Academy of Sciences, Guangzhou, PR China; Key Laboratory of Horticultural Plant Biology (MOE), CHINA

## Abstract

Protoplast isolation is a stress-inducing process, during which a variety of physiological and molecular alterations take place. Such stress response affects the expression of totipotency of cultured protoplasts. MicroRNAs (miRNAs) play important roles in plant growth, development and stress responses. However, the underlying mechanism of miRNAs involved in the protoplast totipotency remains unclear. In this study, high-throughput sequencing technology was used to sequence two populations of small RNA from calli and callus-derived protoplasts in *Citrus reticulata* Blanco. A total of 67 known miRNAs from 35 families and 277 novel miRNAs were identified. Among these miRNAs, 18 known miRNAs and 64 novel miRNAs were identified by differentially expressed miRNAs (DEMs) analysis. The expression patterns of the eight DEMs were verified by qRT-PCR. Target prediction showed most targets of the miRNAs were transcription factors. The expression levels of half targets showed a negative correlation to those of the miRNAs. Furthermore, the physiological analysis showed high levels of antioxidant activities in isolated protoplasts. In short, our results indicated that miRNAs may play important roles in protoplast-isolation response.

## Introduction

MicroRNAs (miRNAs) are a class of endogenous, small non-coding RNAs with length of 20–24 nucleotides. MiRNAs have been demonstrated to negatively regulate gene expression at post-transcriptional level by direct transcript cleavage or translational repression [1–3]. Since the first miRNA, lin-4, was discovered in *Caenorhabditis elegans* [4], lots of miRNAs have been identified in animals, plants, and viruses. According to the miRNAs database (miRBase 21, July 2014), 8,496 mature miRNAs were found in 73 plant species including 53 dicotyledons, 12 monocotyledons, 4 conifers, *Chlamydomonas reinhardtii*, *Physcomitrella patens*, *Selaginella moellendorffii*, and *Amborella trichopoda*. Increasing evidence suggest that plant miRNAs play important roles in all almost biological and metabolic processes, including leaf development [5], shoot branching [6], root growth [7–9], control of flowering time and floral organ identity [10–12], fruit development [13], developmental phase transitions [14–16], auxin signaling [17], programmed cell death [18], and response to biotic or abiotic stresses [19–21].

miRNA identification can be conducted through experimental and/or computational approaches, such as direct cloning, deep sequencing, computational method, homologue-based analysis [20]. Of those, the deep sequencing approach can genome-widely identify known/novel miRNAs due to its ability to quickly produce millions of reads with a determined length [22]. Additionally, because of its advantages in high throughput, high accuracy, and less sample, the deep sequencing has been widely used in many plants, such as *Arabidopsis thaliana* [23, 24], *Citrus sinensis* [25], *Cucumis sativus* [26], *Medicago truncatula* [27], *Oryza sativa* [28], and *Populus trichocarpa* [29]. In citrus, deep sequencing studies has been reported in *Citrus grandis* [30], *Citrus reticulata* [31, 32], *Citrus sinensis* [25, 33–35], and *Citrus trifoliate* [36].

Protoplasts are spherical naked cells obtained after removal of the cell wall usually by enzymatic digestion. Because protoplasts have the characteristics of homogeneous populations, no cell wall, and totipotency, they have been widely used in fundamental research and plant genetic improvement, such as cell wall synthesis, cell division, membrane function, dedifferentiation, somatic hybridization, and transformation [37, 38]. The prerequisite of the protoplast-based technique, especially for plant genetic improvement research, is that protoplasts can regenerate to produce whole plants. However, recalcitrance to regeneration has been observed for protoplasts from most of the agriculturally important plant species, such as rice and grape [39]. The reasons for the protoplast recalcitrance remain largely unknown. Thus, increasing efforts have been paid to decipher the underlying mechanisms of protoplast totipotency.

Expression of plant protoplast totipotency involves protoplast isolation, culture and plant regeneration. During this process, protoplast isolation result in the morphological, physiological or molecular alterations. The incubation time for protoplast isolation varies from several hours to one day, depending on the plant species and the explant. The long-time treatment disrupts cellular redox homeostasis further resulting in oxidative stress. Previous study revealed that ROS were generated during enzymatic isolation of oat mesophyll protoplasts [40]. Thereafter ROS accumulation was also reported in isolated protoplasts of other plants, such as grapevine, tobacco, rapeseed, and rice [41–43]. Furthermore, increased activity of antioxidant machinery was observed in isolated protoplasts of most plants [40, 41, 44, 45]. Thus, such physiological response to protoplast-isolation might be related to regeneration potential of protoplasts. Recently, researchers have paid much attention to the underlying molecular mechanism in the expression of plant protoplast totipotency. The protoplast isolation stage was accompanied by global chromatin decondensation [46–48] and by broad transcriptional and proteomic changes [49–53]. These studies identified a number of differentially expressed genes in isolated protoplasts, such as commonly deregulated transcription factors (TFs), heat shock factor A2, MYB domain protein 7, bZIP63, etc [53]. Such TFs are useful candidates for further investigating the molecular mechanisms underlying plant protoplast totipotency.

Considering the changes in gene expression during protoplast isolation, we hypothesized that miRNAs might play an important role in the regulation of response to protoplast isolation. Nevertheless, to the best of our knowledge, no efforts have been taken to explore protoplast-isolation responsive microRNAs in plants. In this study, attempts were made to identify protoplast-isolation responsive miRNAs in *Citrus reticulate*. Additionally, the physiological response to protoplast-isolation was characterized by the detection of H_2_O_2_ and MDA levels, and analysis of antioxidant enzyme activity (SOD and POD). Our results should provide a foundation for further study of the miRNAs associated with protoplast isolation and uncover the mechanism of protoplast totipotency at miRNA level.

## Materials and methods

### Plant materials

Embryogenic calli of Ponkan mandarin (*Citrus reticulata* Blanco) were maintained on solid MT basal medium supplemented with 50 g L^-1^ sucrose and 7 g L^-1^ agar (pH 5.8). The cell suspensions were established by culturing the calli in liquid MT containing 0.5 g l^-1^ malt extract, 1.5 g l^-1^ glutamine and 50 g l^-1^ sucrose (pH 5.8). After subculture for four cycles at 12-day intervals, the cell suspensions were used for protoplast isolation.

### Isolation of protoplasts

Protoplasts were isolated as previously described [54]. The calli were incubated for 16 hours in an enzyme solution containing 1.5% Cellulase Onozuka R-10, 2% Macerozyme R-10 and 0.7 M EME medium. The isolated protoplasts were purified by passing through 100 and 325 mesh stainless steel sieves and then centrifuged in 26% sucrose and 13% mannitol gradient for 2 min at 910 rpm. The protoplasts were then adjusted to an appropriate density (2–4×10^6^ ml^-1^) in liquid MT supplemented with 40 mg l^-1^ adenine, 0.15 M sucrose, and 0.45 M mannitol.

### Measurement of H_2_O_2_ and malondialdehyde (MDA)

H_2_O_2_ and MDA levels in the calli or protoplasts were evaluated with corresponding assay kits (Nanjing Jiancheng Bioengineering Institute, China). H_2_O_2_ assay was based on the oxidative polymerization of molybdic acid to a complex compound, which can be quantified at 405 nm. MDA assay was based on the thiobarbituric acid (TBA) method and calculated by the absorbance of TBA reactive substances at 532 nm. The absorbance of reaction was read in a spectrophotometer (7210, China). The H_2_O_2_ or MDA levels were expressed as mmol g^−1^ protein or nmol mg^−1^ protein, respectively.

### Analysis of antioxidant enzyme activity

The activity of two antioxidant enzymes, including superoxide dismutase (SOD, EC 1.15.1.1) and peroxidase (POD, EC 1.11.1.7), was determined using assay kits (Nanjing Jiancheng Bioengineering Institute, China), based on the instructions provided by the manufactuerer. The antioxidant enzyme activity was expressed as unit mg^-1^ protein. One unit of SOD was defined as the enzyme activity which inhibit the reduction of NBT to blue formazan by 50%. One unit of POD activity was defined as the amount of enzyme needed to catalyse 1 μg substrate min^-1^ mg^-1^ total proteins present in the homogenate.

### RNA isolation and small RNA sequencing

Total RNAs were extracted from the calli and callus-derived protoplasts using the Trizol regent separately according to the manufacturer’s instructions. The RNA quality was examined by gel electrophoresis and Bioanalyzer (Agilent2100). Small RNAs with 16-30nt in length were separated from the total RNAs by size fractionation with polyacrylamide gel electrophoresis. After PAGE purification and ligation of adaptors to their 5' and 3' ends, the small RNAs were reverse transcribed to cDNA using Superscript Ⅱreverse transcriptase (Invitrogen). The amplified products were purified by gel electrophoresis and sequenced via the Illumina Genome Analyzer at the Beijing Genomics Institute (BGI, Shenzhen, China). All small RNA sequencing data presented in this study is available from the NCBI database (accession number(s) SRP106981).

### Bioinformatic analysis for miRNA identification

To identify the conserved and novel miRNAs in *Citrus reticulata*, the raw sequences were pre-processed as described by literature [55]. After removal of the poor-quality reads and adaptor sequences, high quality trimmed sequences from18 to 30nt were used for further analysis. Initially, rRNA, tRNA, snRNA, snoRNA and poly-A tail were removed from among the small RNA sequences. The remaining sequences were compared with the citrus sequence deposited in GenBank (http://www.ncbi.nlm.nih.gov/genbank) and Rfam database (http://www.sanger.ac.uk/software/Rfam). Then, the remaining sequences were used to perform a BLASTN search against the known plant miRNAs in the miRNA database (http://www.mirbase.org/), only the perfectly matched sequences were considered to be conserved miRNAs. Reads that were not annotated were used to predict novel miRNAs by Mireap software(https://sourceforge.net/projects/mireap), which was developed by the BGI. The novel miRNAs were identified through the criterion previously developed for plant miRNA identification by literature [56].

### Differential expression analyses of miRNAs

The miRNA expression between two samples (calli and protoplasts) was compared to sort out differentially expressed miRNAs (DEMs). The count of each miRNA was normalized to transcript per million (TPM). Normalized expression was calculated by the following formula:the normalized expression = miRNA expression/ sample total expression×order of magnitude. The fold-change between calli and protoplasts was calculated by the following formula: Fold-change = log2 (protoplasts / calli). The miRNAs with fold-change >1 or <-1 and p-value ≦ 0.01 were considered upregulated or downregulated in response to protoplast-isolation, respectivedly. The p-value was calculated by the literature [35]

### Target prediction of miRNAs

Target predictions were performed based on the criteria suggested by literatures [57, 58]. The criteria were set as follows: (1) The mismatch between sRNA and target gene should not exceed 4 (G-U bases count as 0.5 mismatchs); (2) There should be no more than 2 adjacent site mismatchs in the miRNA/target duplex; (3) In the miRNA/target duplex, 2–12 locus of 5 'end of miRNA must not have adjacent mismatches; (4) The 10–11 site of miRNA/target duplex must have no mismatches; (5) In the miRNA/target duplex, 1–12 locus of 5 'end of miRNA must not have more than 2.5 mismatches; (6) The minimum free energy (MFE) of miRNA/target duplex should not be less than the 75% MFE of miRNA bound to its exact complement.

### Validation of miRNA expression using qRT-PCR

The analysis of miRNA expression was performed using poly(A)-tailed real-time RT-PCR method as described in literature [59]. Firstly, Small RNA was isolated from calli and protoplasts with RNAiso for Small RNA(TaKaRa) according to the manufacturer’s instructions. Then the Small RNA were polyadenylated by poly(A) polymerase, and recovered with phenol/chloroform extraction and ethanol precipitation. Subsequently, the RNA was reverse-transcribed into cDNA with poly(T) adapter according to PrimeScript^TM^ 1st strand cDNA synthesis Kit and then the qRT-PCR was performed on a BIO-RAD CFX96TM Real-Time System with the SYBR®Premix Ex TaqTM II (Tli RNaseH Plus) (TaKaRa) according to the manufacturers’s instructions. The 5.8S rRNA gene was used as the reference gene. All primer sequences were listed in S1 Table. The conditions for the PCR amplification were as follows:45 cycles of 95°C for 30 s, 95°C for 10 s, 60°C for 15 s, and 72°C for 20 s. The relative expression ratio was calculated using the ΔΔCt method.

### Semi-quantitative RT-PCR analysis of miRNA target genes

Total RNAs were extracted as above description. The RNA samples were used for cDNA synthesis using PrimeScript^TM^ 1st Strand cDNA Synthesis Kit following the manufacturer’s instructions. The primer sequences of target genes were listed in S2 Table. The conditions for the PCR amplification were as follows: 94°C, 5min; 30 cycles of 94°C, 30 s, 55–65°C, 30 s and 72°C, 60s; at last, 72°C, 8 min. The same cDNA was amplified with primers specific to an actin gene, which was used as an internal positive control.

### Statistical analysis

The experiments were repeated at least 3 times with 3 replicates for each. All data, expressed as mean ± SD, were analyzed using SPSS 17.0 software (SPSS Inc., Chicago, IL). A comparison between groups was conducted by analysis of variance (ANOVA).

## Results

### Physiological response to protoplast-isolation

**Fig 1:**
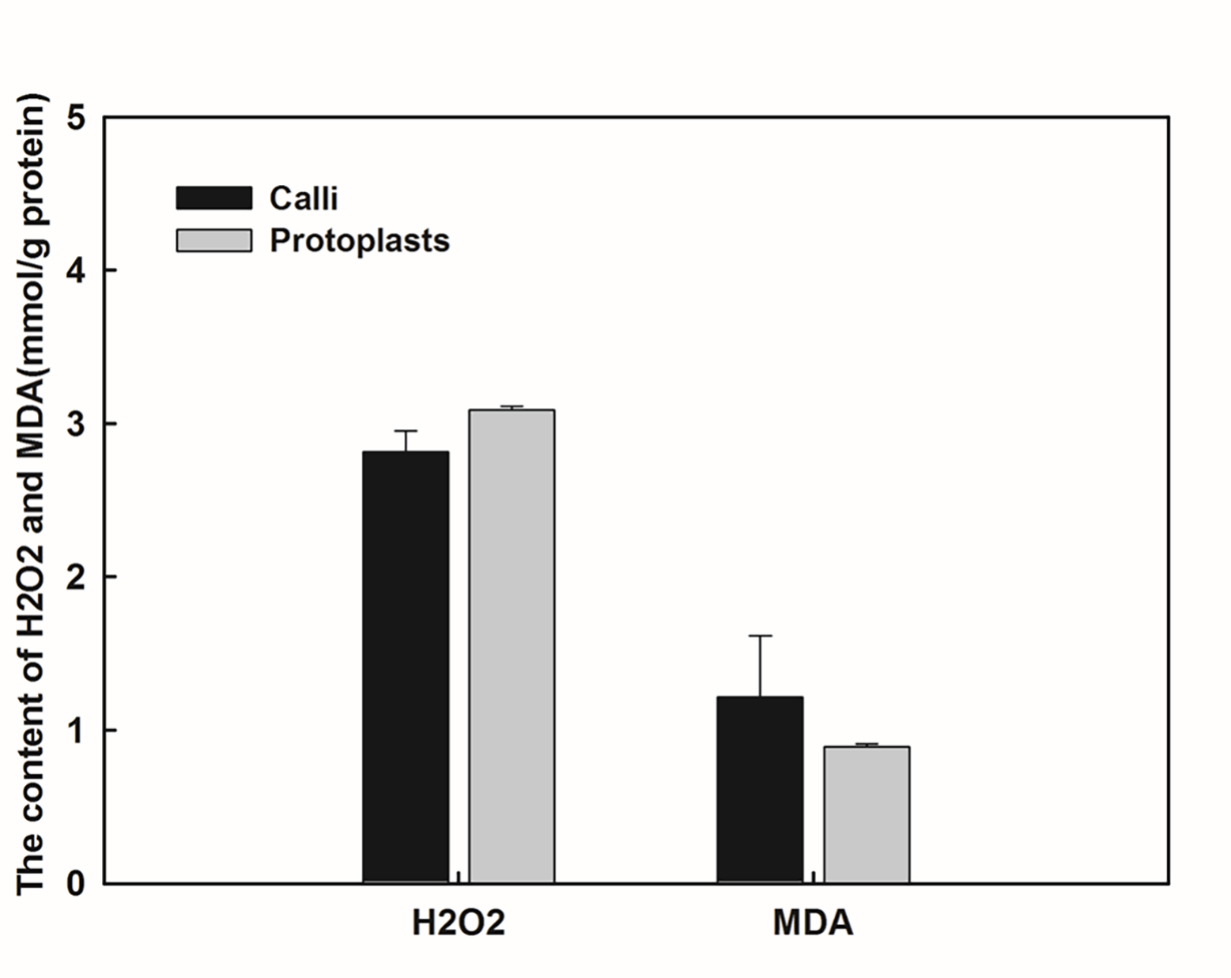
Assessment of H_2_O_2_ and MDA levels in calli and protoplasts. The data were presented as mean±SD of three biological repeats. ANOVA were used to statistical analysis and no significant difference between calli and protoplasts was detected.

**Fig 2:**
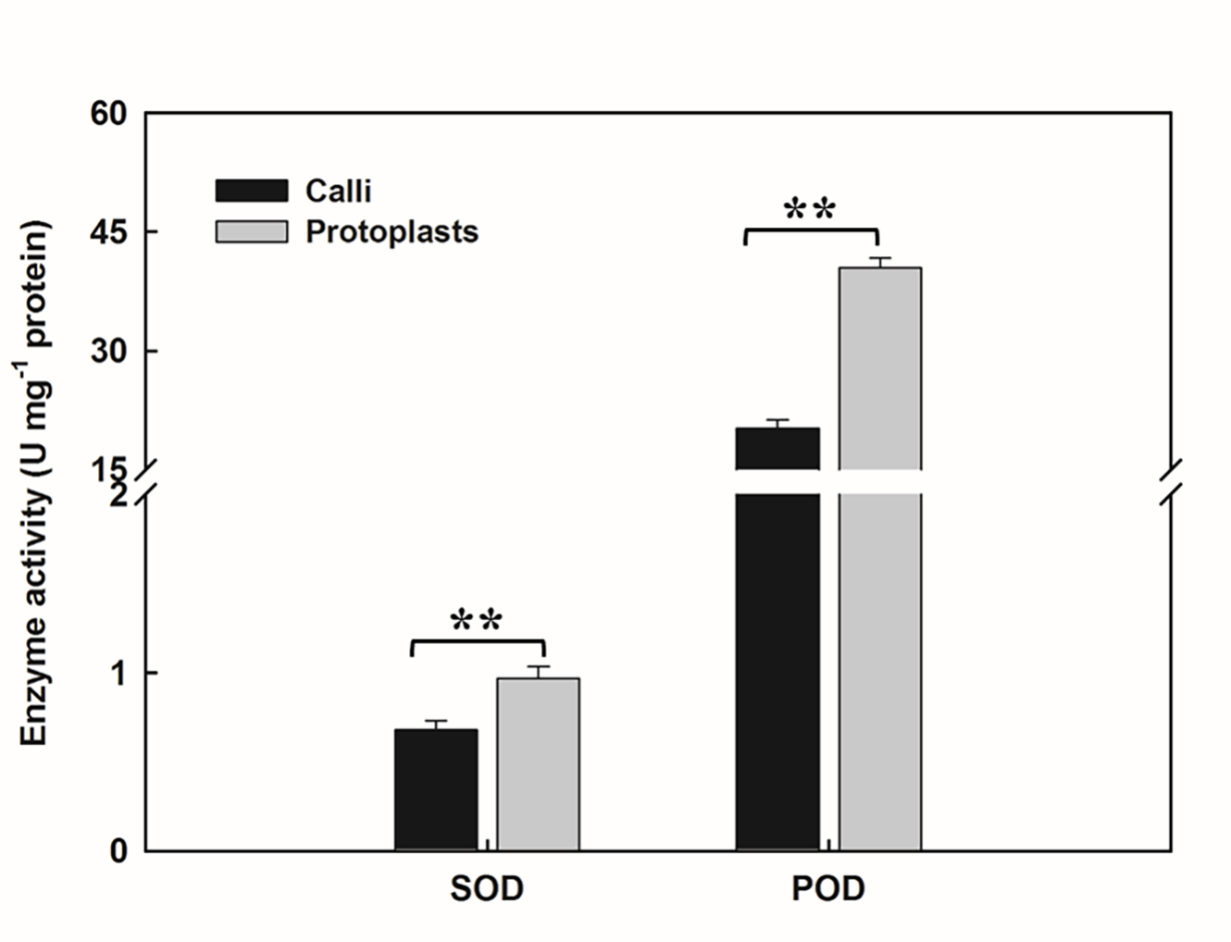
Evaluation of the activity of POD and SOD in calli and protoplasts. The data were presented as mean±SD of three biological repeats. ANOVA were used to statistical analysis and * * indicate the significant difference at *P* < 0.01 level.

The regeneration potency of protoplasts was demonstrated to be related to the physiological status, especially with magnitude of ROS production and removal[39, 54]. In this study, we detected H_2_O_2_, MDA and antioxidant enzyme activity levels in calli and callus-derived protoplasts (Figs 1 and 2). After removal of cell wall, H_2_O_2_ levels of the isolated protoplasts displayed a slight increase (9.67%); and MDA levels exhibited a little decrease (26.91%) although there were no statistical significant difference. SOD activity in the protoplasts was 1.42 times higher than that in the calli, while the protoplasts had 2.0-fold higher POD activity compared with the calli.

### Small RNA library construction and sequencing

**Fig 3:**
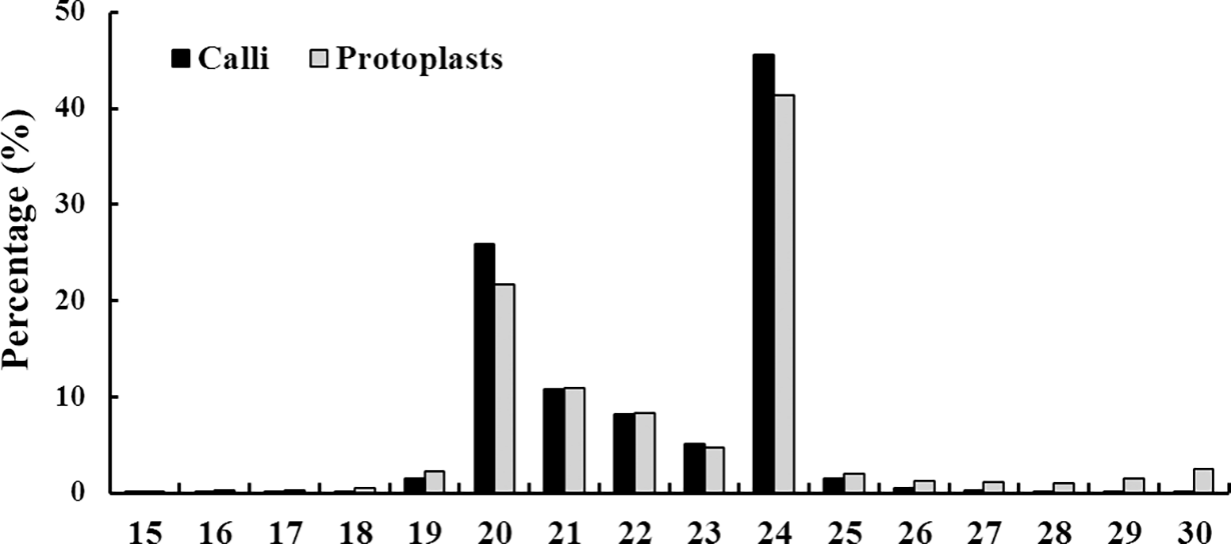
Length distributions of small RNAs in the calli and protoplasts.

To identify protoplast-isolation responsive microRNAs, we constructed two small RNA libraries, namely, callus library and protoplast library. After sequencing, 17,975,734 and 15,828,591 total reads were obtained from callus library and protoplast library, respectively. These total reads were cleaned by removing adaptors, contaminants, and low quality tags, thus generating 17,899,040 (99.74%) and 15,663,034 (99.22%) clean reads including miRNA, rRNA, snRNA, snoRNA, tRNA, degraded fragments of mRNA introns or exons and several other unannotated reads (Table 1). Additionally, 2,303,525 (38.76%) unique reads from calli and 2,017,839 (39.30%) unique reads from protoplasts were mapped to orange genome using SOAP software. The length of the sRNAs varied from 15 to 30 nt, with 24 nt being the most abundant, followed by 20 nt, 21 nt and 22 nt (Fig 3). Removal of cell wall resulted in fewer 20 nt and 24 nt sRNAs and more 21 nt and 22 nt sRNAs.

**Table 1:** Summary of small RNAs from calli and protoplasts of *Citrus reticulate*.

Category	calli	protoplasts
Total reads	17,975,734	15,828,591
High quality	17,945,812	15,786,575
3’ adaptor null	11,146	10,665
Insert null	700	1,436
5’ adaptor contaminants	9,082	1,0284
Smaller than 18 nt	23,368	98,740
Poly A	2,476	2,416
Clean reads	17,899,040 (99.74%)	15,663,034 (99.22%)
Total sRNAs	17,899,040	15,663,034
Unique sRNAs	5,943,092	5,134,879
Mapped to genome	2,303,525 (38.76%)	2,017,839 (39.30%)
Exon-antisense	136,263 (2.29%)	124,857 (2.43%)
Exon-sense	207,389 (3.49%)	189,202 (3.68%)
Intron-antisense	48,642 (0.82%)	43,348 (0.84%)
Intron-sense	61,032 (1.03%)	54,931 (1.07%)
miRNA	655 (0.01%)	633 (0.01%)
rRNA	47,046 (0.79%)	49,911 (0.97%)
snRNA	1,783 (0.03%)	2,848 (0.06%)
snoRA	1,360 (0.02%)	2,405 (0.05%)
tRNA	7,849 (0.13%)	12,600 (0.25%)
Unann	5,431,073 (91.38%)	4,654,144 (90.64%)

### Identification of known and novel miRNAs

To identify the known miRNAs, clean reads were mapped with miRBase. A total of 67 known miRNAs belonging to 35 families were identified in the two libraries (S3 Table). The 35 known miRNA families consisted of 23 conserved and 12 non-conserved miRNA families. Most of the conserved miRNA families have multiple members. Among them, the miR166 family with nine members was the largest conserved miRNA family, followed by the miR167 and miR171 family. However, the non-conserved miRNA families had only one member. In addition, the count of reads was normalized to transcript per million (TPM) in order to compare the abundance of miRNAs in the two libraries. The most abundant miRNA was miR156 (207387.83 TPM), followed by miR3954 (3236.04 TPM). This indicates that the two miRNAs may play an important role in the callus growth. A total of 277 novel miRNAs were identified in the two libraries using Mireap software (S4 Table). Among them, these were 104 and 85 specific miRNAs in calli and protoplasts, respectively. Additionally, about 90% novel miRNAs are in low relative abundance less than10 TPM.

### Identification of differentially expressed miRNAs (DEMs) in callus and protoplast

To uncover the potential role of miRNA in the protoplast-isolation response, DEM were identified. DEM was defined with >1 fold change in expression level and *P* value < 0.01. Based on these criteria, a total of 18 known miRNAs were shown to be response to protoplast-isolation: 12 up-regulated and 6 down-regulated (Table 2). Meantime, a total of 64 novel miRNAs showed differential expression: 29 up-regulated and 35 down-regulated (S5 Table). The strongest up-regulated known (novel) and down-regulated known (novel) miRNAs were crt-miR482b with a fold-change of 3.08 (novel_mir_262 with a fold-change of 10.83) and crt-miR166e* with a fold-change of -2.46 (novel_mir_35 with a fold-change of -13.31), respectively.

**Table 2:** Characterization of differentially expressed miRNAs in response to protoplast-isolation.

miRNA	Normalized count (TPM)		Fold change	p-value
	callus	protoplast		
**Up-regulated miRNAs**				
crt-miR396a	73.91	148.57	1.01	1.71E-95
crt-miR396b	74.42	154.95	1.06	8.66E-108
crt-miR171.3	20.00	49.48	1.31	2.91E-49
crt-miR408	22.29	57.46	1.37	7.64E-61
crt-miR482a	18.83	55.29	1.55	8.49E-71
crt-miR172a	10.67	31.48	1.56	2.71E-41
crt-miR171a	0.56	1.66	1.57	0.002
crt-miR171.2	0.56	1.66	1.57	0.002
crt-miR167c	45.70	199.83	2.13	0
crt-miR827	11.90	53.57	2.17	1.01E-107
crt-miR472	28.66	132.61	2.21	6.55E-270
crt-miR482b	5.14	43.41	3.08	1.03E-129
**Down-regulated miRNAs**				
crt-miR166e*	20.34	3.70	-2.46	5.20E-47
crt-miR319.2	2.12	0.57	-1.89	0.0001
crt-miR172a*	2.57	0.70	-1.87	2.05E-05
crt-miR482a*	88.33	28.16	-1.65	4.09E-119
crt-miR535	171.63	65.19	-1.40	2.48E-180
crt-miR319.1	3.97	1.72	-1.20	0.0001

### miRNA target gene prediction and functional annotation analysis

Identification of miRNA targets is important for the understanding of miRNA functions [60]. To better illustrate protoplast-isolation responsive microRNAs in citrus, the target genes of DEMs were predicted. A total of 56 (141) target genes were identified based on the 16 (38) differentially expressed known (novel) miRNA in the present study (S6 and S7 Tables). Whereas, 28 DEMs had no predicted target genes. For known miRNAs, a large number of target genes were transcription factors, such as GRAS (crt-miR171a, crt-miR171.2, crt-miR171.3), APETALA 2 (crt-miR172a), Auxin response factor (crt-miR167c), MYB (crt-miR827), TCP2 (crt-miR319.1, crt-miR319.2), and GAMYB (crt-miR319.1, crt-miR319.2). Interestingly, some target genes were involved in the anti-disease response, such as LRR and NB-ARC domain-containing disease resistance protein genes (crt-miR482b). For novel miRNAs, most of the identified target genes were annotated to be involved in different biological and molecular processes, which also included some anti-disease genes.

### Analysis of the expression of differentially expressed miRNAs and their target genes

**Fig 4:**
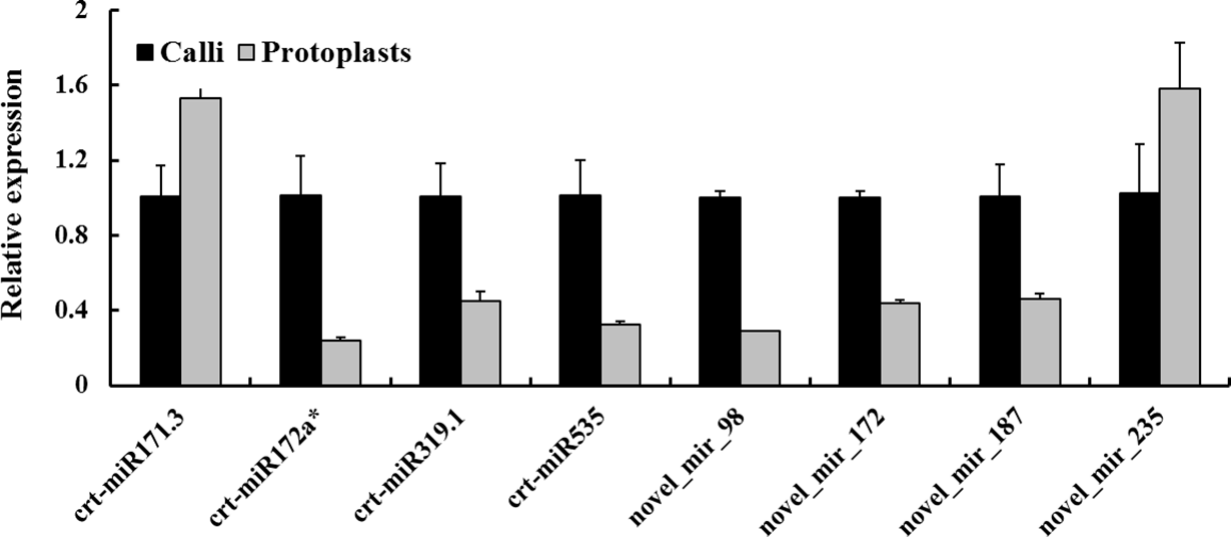
qRT-PCR analysis of miRNAs expression levels in calli and protoplasts.

**Fig 5:**
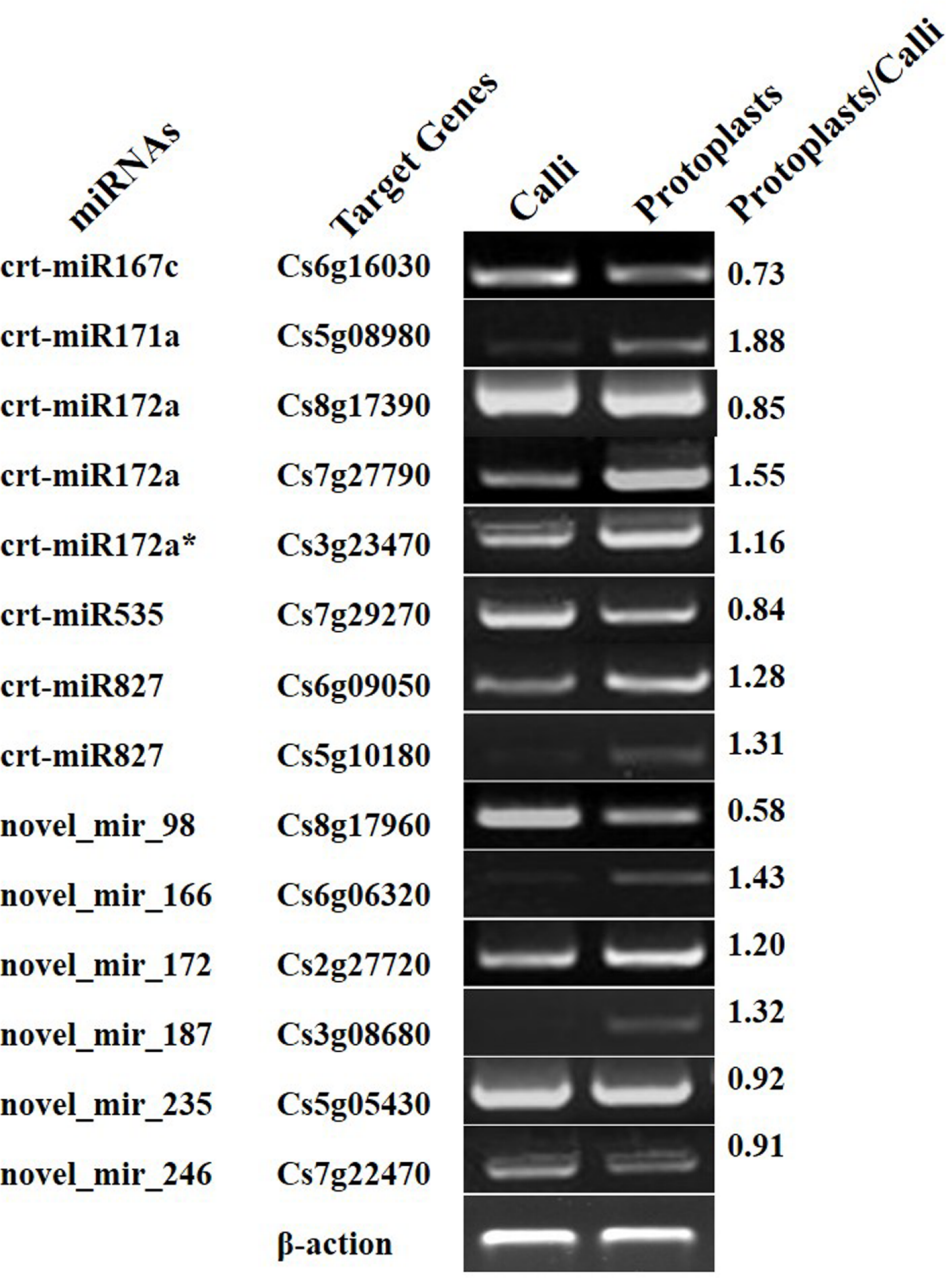
The expression levels of miRNA target genes in calli and protoplast using semi-quantitative RT-PCR. The gene expression ratios of protoplast to calli were analyzed with ImageJ software.

To confirm the results obtained by deep sequencing, qRT-PCR was performed to examine the expression profiles of eight DEMs (crt-miR171.3, crt-miR172a*, crt-miR319.1, crt-miR535, novel_mir_98, novel_mir_172, novel_mir_187, and novel_mir_235). As shown in Fig 4, crt-miR171.3 and novel_mir_235 showed an up-regulated pattern; other miRNAs showed down-regulated pattern. Clearly, the results of qRT-PCR analysis were consistent with those of deep sequencing although the variation of the absolute fold change was found between the two techniques. The transcription levels of 14 genes (Cs6g16030, Cs5g08980, Cs8g17390, Cs7g27790, Cs3g23470, Cs7g29270, Cs6g09050, Cs5g10180, Cs8g17960, Cs6g06320, Cs2g27720, Cs3g08680, Cs5g05430, and Cs7g22470) targeted by 7 up-regulated miRNAs and 5 down-regulated miRNAs were detected by semi-quantitative RT-PCR. As shown in Fig 5, 7 (50%) target genes displayed expected changes, which indicated that the expression patterns of these target genes were negatively regulated by miRNAs.

## Discussion

Our previous study showed that the callus-derived protoplasts of *Citrus reticulata* exhibited high levels of antioxidant activities during in vitro culture [54]. However, the physiological response to protoplast-isolation is still unknown. In this study, after detection of H_2_O_2_ and MDA levels, and analysis of antioxidant enzyme activity (SOD and POD), we found the isolation of protoplast led to increased antioxidant levels and less robust oxidative stress. Similar results were observed in grapevine and tobacco [39]. These results suggest that the removal of cell wall can activate antioxidant systems and such physiological status was maintained in the time course of culture period.

In our research, the sRNA sequences from callus and protoplast libraries showed a wide variation in length, ranging from 18 to 30 nt, of which the most abundant sRNAs were 24 nt, accounted for 45.62% and 41.38% of all sRNAs, respectively (Fig 3). The result was consistent with previous findings in sRNAs from calli in sweet orange, cotton, sugarcane, and rice [61–64], which imply that this sRNA profiling is distributed in a tissue-dependent manner. Interestingly, with regard to the sRNA sequences from various tissues and organs in sweet orange, 24 nt was the predominant type; but for other types of citrus, 21 nt was the highest [25, 30–34, 36, 64, 65]. In plants, sRNAs were produced by DCL enzymes that are key determinants of sRNA size, and DCL3 produces the 24-nt sRNAs [66]. Therefore, the sRNAs from the callus and protoplast libraries are speculated to be produced mainly by DCL3. It is worth noting that the size distribution of miRNAs was almost at 20–23 nt (S3 and S4 Tables). Thus the 24-nt sRNAs mainly consisted of small interfering RNA (siRNA), which are involved in histone and DNA modifications that result in transcriptional gene silencing [67].

In citrus, 79 miRNAs, which contained 64 miRNAs in *Citrus sinensis*, 6 miRNAs in *Citrus trifoliate*, 5 miRNAs in *Citrus clementine*, and 4 miRNAs in *Citrus reticulate*, were annotated in the latest version miRBase 21[68, 69]. Here, we identified a total of 67 known miRNAs (18 DEMs) and 277 novel miRNAs (64 DEMs) from callus and protoplast libraries, suggesting that miRNAs play important roles in citrus protoplast isolation. In contrast with the known miRNAs, the novel miRNAs have lower abundance and bigger fold-change (Table 2, S5 Table), indicating that the known miRNAs are probably responsible for controlling the basic cellular and developmental processes, but the novel miRNAs are involved in the regulation of the specific regulatory pathways and functions [70]. For example, the most abundant miRNA was miR156 in our study, which is also one of the most abundant and evolutionarily conserved miRNAs in plants. MiR156, which targets a series of *SQUAMOSA PROMOTER BINDING PROTEIN-LIKE* (*SPL*) genes, controls many aspects of plant development and physiology by regulating SPL levels, such as change of flavonol content in Arabidopsis [71]. Recently, the miR156-SPL module was reported to regulate starch accumulation in citrus callus, and overexpression of miR156 resulted in significantly higher content of starch [72]. Thus, the observed down-regulation of miR156 in protoplast isolation might result in high levels of flavonols and starch degradation, which can protect the isolated protoplasts from oxidative stress.

The protoplast isolation *per se* is a stress-inducing procedure [39], because the cells are subjected to enzymatic stress induced by cellulose/macerozyme and osmotic stress from the osmotic solution. Thereby, many researchers have found that most differentially expressed genes were associated with stress responses during protoplast isolation from moss, cotton, Arabidopsis, or rice [49, 50, 52, 53]. It is well known that some miRNAs play critical roles in stress responses [19–21], such as miR156, miR172, miR319, miR396, miR408, miR482, and miR535. In citrus protoplast isolation, these miRNAs were all detected (Table 2). This suggests that the miRNAs are considered to be involved in the regulation of response to protoplast isolation.

MiR319 is one of the first characterized and conserved miRNA families in plants and was found to regulate plant development and abiotic/biotic stress response by targeting *TEOSINTE BRANCHED/CYCLOIDEA/PROLIFERATING CELL FACTORS* (*TCP*) genes [73]. Transgenic creeping bentgrass overexpressing Osa-miR319a was reported to exhibit enhanced drought and salt tolerance [74]. And the induction of miR319 expression during cold stress was observed in sugarcane [75]. However, in tomato miR319 overexpression reduces resistance to root-knot nematode, and miR319 negatively regulates RKN resistance by targeting *TCP* genes and affecting the jasmonic acid (JA) level [76]. Therefore, the observed down-regulation of miR319 in citrus protoplasts indicated that enzymatic stress is similar to abiotic stress, and positive response to protoplast-isolation may be related with miR319/TCP module. In the future, the expression levels of all *TCP* genes and JA levels will be determined in the citrus calli and protoplasts. And the role of miR319 in protoplast-isolation response needs to be tested by its over-expression or silencing.

MiR482 has been demonstrated to be the trigger for production of phasiRNAs from their target transcripts of disease resistance proteins that included many *NBS-LRR* genes [77]. In our study, different members of miR482 family showed different expression patterns. For instance, crt-miR482a and crt-miR482b were up-regulated, but crt-miR482a* was decreased during protoplast-isolation. Surprisingly, many novel miRNAs which targeted disease resistance genes showed an up or down-regulated change, such as novel_mir_25, novel_mir_27, novel_mir_44, novel_mir_178, and novel_mir_54. These results suggest that disease resistance genes play an important role in protoplast-isolation response. In embryogenic tissues from ‘Valencia’ sweet orange, the disease resistance genes were also found to be derepressed through miRNA-mediated regulation [64]. In short, positive stress response, such as downregulated miR319 or miR482, may be regarded as one of the key indicator for expression of plant protoplast totipotency.

In conclusion, our study provides a comprehensive identification of miRNAs between calli and callus-derived protoplasts in *Citrus reticulata* Blanco. A total of 67 known miRNAs and 277 novel miRNAs were identified. And the expression of miRNAs was greatly altered, such as miR156, miR319 and miR482, which may be related to protoplast totipotency. The miRNAs are considered to be involved in the protoplast-isolation response. Additionally, in physiological level the removal of cell wall can activate antioxidant systems.

## Supporting information

S1 Table Primer list of miRNAs qRT-PCR used in this study. (DOC)

S2 Table Primer list of semi-quantitative RT-PCR used to evaluate the expression level of miRNA target genes. (DOC)

S3 Table Characterization of known miRNAs in calli and protoplasts. (XLS)

S4 Table Characterization of novel miRNAs in calli and protoplasts. (XLS)

S5 Table Differentially expressed novel miRNAs in response to protoplast-isolation. (XLS)

S6 Table Target genes of known miRNAs in calli and protoplasts. (XLS)

S7 Table Target genes of novel miRNAs in calli and protoplasts. (XLS)
