# Supplementary material for: Identification of protoplast-isolation responsive microRNAs in Citrus reticulata Blanco by high-throughput sequencing
Source: PLoS One. 2017 Aug 22;12(8):e0183524. doi: 10.1371/journal.pone.0183524 (PMC5567906; doi:10.1371/journal.pone.0183524)
Supplement: S1 Table — (DOC) [file pone.0183524.s001.doc]

**Table S1. Primer list of miRNAs qRT-PCR used in this study**

| *Gene name* | *miRNA sequences* | *Forward primer (5´→3´)* | *Reverse primer (5´→3´)* |
| --- | --- | --- | --- |
| crt-miR171.3 | UGAUUGAGCCGUGCCAAUAUC | ATTCTAGAGGCCGAGGCG | TGATTGAGCCGTGCCAATATC |
| crt-miR172a* | GCAGCGUCCUCAAGAUUCACA | ATTCTAGAGGCCGAGGCG | GCAGCGTCCTCAAGATTCACA |
| crt-miR319.1 | UUUGGACUGAAGGGAGCUCCU | ATTCTAGAGGCCGAGGCG | TTTGGACTGAAGGGAGCTCCT |
| crt-miR535 | UGACAAUGAGAGAGAGCACAC | ATTCTAGAGGCCGAGGCG | TGACAATGAGAGAGAGCACAC |
| novel_mir_98 | AGCUGAUGAUGAGAAACAUUUA | ATTCTAGAGGCCGAGGCG | AGCTGATGATGAGAAACATTTA |
| novel_mir_172 | CAAUAAAAAACUUGUGGUGAUGU | ATTCTAGAGGCCGAGGCG | CAATAAAAAACTTGTGGTGATGT |
| novel_mir_187 | UGGAUCAUGUUGAGGCUUCAC | ATTCTAGAGGCCGAGGCG | TGGATCATGTTGAGGCTTCAC |
| novel_mir_235 | CUUUCAGCAGCCUCCGGCGUC | ATTCTAGAGGCCGAGGCG | CTTTCAGCAGCCTCCGGCGTC |
| 5.8S rRNA |  | CTCGGCAACGGATATCTCGGCTCT | CTAATGGCTTGGGGCGCAACTTG |
