# Supplementary material for: Identification of protoplast-isolation responsive microRNAs in Citrus reticulata Blanco by high-throughput sequencing
Source: PLoS One. 2017 Aug 22;12(8):e0183524. doi: 10.1371/journal.pone.0183524 (PMC5567906; doi:10.1371/journal.pone.0183524)
Supplement: S2 Table — (DOC) [file pone.0183524.s002.doc]

**Table S2. Primer list of semi-quantitative RT-PCR used to evaluate the expression level of miRNA target genes.**

| **miRNA** | **Target gene** | **Forward primer (5´→3´)** | **Reverse primer (5´→3´)** |
| --- | --- | --- | --- |
| crt-miR167c  crt-miR171a  crt-miR172a  crt-miR172a | Cs6g16030.1  Cs5g08980.1  Cs8g17390.1  Cs7g27790.1 | TACTCACAAATGCCTCCC  CTGCCAACAGAGGACAAG  ATTTGCCATGCCCAGTTA  CATTTGGCAAGCCTCACA | TGAAACGGATGAAGTGTATG  CTCAAGGCAAATGGAAAC  AGTCGGCTCGGTCGTATT  AGGCTGTTCCAATCACGC |
| crt-miR172a* | Cs3g23470.1 | GCATCCATTGTTCGGTTT | AGCATCGTTGCCAATACA |
| crt-miR535 | Cs7g29270.1 | ACTCCAAACGGGACATAA | CGACGCTCTTTCCATACT |
| crt-miR827  crt-miR827 | Cs6g09050.1  Cs5g10180.1 | TTAGGTGGAACTGGCTCA  GGAAGACATCACCCGAGA | AGCAGATGGAAGGGTTGA  TTAGGTGCTGTTGTGGGA |
| novel_mir_98  novel_mir_166 | Cs8g17960.1  Cs6g06320.1 | AAACAGACCAGGGAAAGC  CTGAACTTTCTGGGCTGA | AGAGGACGAGTCACCAATC  CACGGGACTGAATGATGT |
| novel_mir_172 | Cs2g27720.1 | TGTTTCCACTGCCTCAAT | TGACGAGGATTATTTGGC |
| novel_mir_187 | Cs3g08680.1 | GCTGCTAAGACGAGACCC | CATACGGCTCCTACTGGC |
| novel_mir_235 | Cs5g05430.1 | TGGTGGCTCATTGGTTCG | TTTCGCCGTGTTCCTGAC |
| novel_mir_246 | Cs7g22470.1 | GCAGTGCCGACAAAGAAG | CAGGGCAACAGCGACATA |
| β-action |  | GACTCTGGTGATGGTGT | CACTTCATGATGGAGTTGT |
